# Medical Opinion and Movement

**Published:** 1907-12-14

**Authors:** 


### Medical Opinion and Movement.

Alcoholism in France is held to be one of the most terrible scourges of the present day, and is also, apparently, one of the chief causes of death. Two years ago the Académie de Médecine invited members of the medical societies to collect statistics of all deaths in their wards. M. Fernet has communicated a summary of these statistics, and finds that during the last fifteen months, among a total of over 1,500 deaths occurring in eleven different hospitals and asylums, alcohol played a part in the cause of death in one-third of the cases. It was the principal cause in one-tenth of the deaths, and it was the accessory cause in more than two-tenths. As the principal fatal manifestations of alcohol are included such affections as delirium tremens, pachymeningitis hæmorrhagica cirrhosis, and cardio-vascular diseases. Conditions in which alcohol played an accessory part to the fatal issue include cases of pneumonia, erysipelas, and other acute infectious diseases. Alcohol showed a still greater influence on mortality in asylums than in the general hospitals. At the Asile Sainte Anne 30 out of 63 consecutive deaths were among alcoholics. In asylums generally alcohol was the cause of disease and death in nearly half the male cases, and in one-sixth of the female cases. In view of the recent discussions as to the relation of alcohol to insanity in this country these figures are of a special interest.

Some time back Dr. William Bruce drew attention to the relation between sciatica and hip-joint disease, and put forward the view that in a large number of cases, presenting all the symptoms of sciatica, careful examination would reveal some morbid condition of the hip-joint, often of a chronic rheumatoid nature. This proposition has found a further advocate in Dr. W. Ironside Bruce, who has recently delivered a paper on the subject before the Medical Society of London. He has demonstrated the truth of this theory by radiographs, which clearly showed articular changes in five out of twelve cases of sciatica observed by him. He points out that in early cases the inflammatory changes in the joint may be so slight that a radiograph would not clearly demonstrate them. It is a recognised fact that tuberculous disease of the hip-joint is frequently associated with pain, referred to the knee-joint, owing to the identity of nerve supply by the obturator nerve, and it seems highly probable that other inflammatory conditions of the hip-joint would in the same way initiate referred pain in neighbouring parts. Many cases of so-called sciatica are accompanied by wasting of the gluteal muscles, and as these muscles are not supplied by the sciatic nerve, the supposed neuritis of this nerve does not explain the phenomenon. Moreover, it is maintained that many intractable cases of so-called sciatica develop into definite cases of chronic rheumatism of the hip-joint. The deep-seated position of the joint makes an early diagnosis often difficult. Unfortunately when the condition is clearly distinguished and diagnosed, the patient is not much better off, for there are few conditions more intractable to treatment.

Dr. D. C. L. Fitzwilliams, discussing the etiology and pathology of intussusception in children, comes to the conclusion that the dietetic factor plays an important *rôle* in the causation of the disease. His observations are the result of investigations of over 1,000 cases, and he bases his arguments on seasonal incidence, on age and on sex. A curve of incidence made from 453 cases under one year of age shows a marked rise in the early spring, and again in December, and these fluctuations he associates with the festivities of Easter and Christmas, when the infants are especially liable to obtain a share of the tit-bits provided for the rest of the family. Sixty-four per cent. of cases under one year fell between the ages of four to seven months, and it is suggested that this is the period when articles of food unsuited to the age may be forced upon the infant. Moreover, there is a great preponderance of cases in male infants, and this may be accounted for by the fact that male children are larger and stronger, and are, therefore, given more food than those of the opposite sex. The disease is certainly less common in the more well-to-do than in the hospital class of people, who are ignorant of the harm done by improper feeding with solid food. These facts, at any rate, serve to emphasise the importance of educating the poorer classes in proper methods of rearing their infants.

The Huddersfield scheme for the prevention of infantile mortality has been such a complete success that we cannot refrain from again referring to it in these columns, in the hope that other towns may be stimulated to follow the excellent example of this industrial centre. The bulk of the work is carried on by a voluntary association of upwards of 100 ladies, called the Huddersfield and District Public Health Union. This association works in harmony with and under the direction of the Medical Officer of Health, who has two assistant lady Medical Officers of Health specially for the purpose. By a special Act the Corporation has power to require compulsory notification of births within forty-eight hours. Immediately on receipt of the notification one of the lady assistants proceeds to the address, and if help or advice seems advisable, it is tendered, and specially prepared leaflets of advice are left, and the cases are then passed on to the Public Health Union, so that each body is placed under the care of some lady helper. When no medical man is in attendance the lady doctor is called in when further assistance is required, and cases of need are referred to various charitable and philanthropic agencies. As a result of this well-organised scheme the infant mortality figure for the first thirty-nine weeks of 1907 has been eighty-five, as compared with 135 for a corresponding period last year. On comparision with other towns, it appears that for the first six months of the current year Huddersfield has an infant mortality lower by 22 per cent. than the seventy-six great towns, and for the third quarter the infant mortality is lower by 44 per cent. The total cost to the Corporation does not amount to £400 per annum.

